# Supplementation of the Probiotic LLH135 Reduces Oxidative Stress in a Model of Hemiparkinsonism

**DOI:** 10.1155/bn/8401392

**Published:** 2025-04-10

**Authors:** Mario E. Flores-Soto, Angelica Y. Nápoles-Medina, Aldo R. Tejeda-Martínez, Josué R. Solís-Pacheco, Verónica Chaparro-Huerta, Juan E. Gutiérrez-Sevilla, Blanca R. Aguilar-Uscanga

**Affiliations:** ^1^Laboratorio de Neurobiología Celular y Molecular, División de Neurociencias, Centro de Investigación Biomédica de Occidente (CIBO), Instituto Mexicano del Seguro Social, Guadalajara, Jalisco, Mexico; ^2^Laboratorio de Investigación Leche humana, Departamento de Farmacobiología, Centro Universitario de Ciencias Exactas e Ingenierías, Universidad de Guadalajara, Guadalajara, Jalisco, Mexico

**Keywords:** hemiparkinsonism, *Lactobacillus*, oxidative stress, Parkinson's disease, probiotics

## Abstract

Oxidative stress and neuroinflammation are considered as the two main etiological reasons behind idiopathic Parkinson's disease (PD). Nevertheless, the actual treatments are focused on improving motor symptoms by restoring dopamine (DA) presence, leaving said causes unattended. Probiotics could be a promising strategy for the improvement of these physiological features behind the disease and therefore constitute a complementary treatment for those having PD. This study evaluated the effect of the oral administration of a probiotic bacteria mixture from 3 strains of *Limosilactobacillus fermentum* LH01*, Limosilactobacillus reuteri* LH03, and *Lactiplantibacillus plantarum* LH05 (LLH135), of human milk origin, for 4 weeks, on mice under the hemiparkinsonism model of intrastriatal administration of 6-hidroxidopamine (6-OHDA). We measured total antioxidant capacity (TAC), super oxide dismutase (SOD) activity, and 8-deoxyguanosine (8-OHdG) regarding oxidative stress. Concerning neuroinflammation, immunoreactivity for GFAP, IBA-1, and CD68 was measured by immunohistochemistry and the latter markers corroborated in colocalization with immunofluorescence to assess activated microglia. The probiotic mixture diminished the oxidative stress features of SOD activity as well as 8-OHdG generated by the model of hemiparkinsonism. These effects were accompanied as well by the dampening of the glial immunoreactivity and colocalization of IBA-1 and CD68 that were present under the model. Our findings suggest that the administration of the probiotic LLH135 exerts neuroprotective effects by promoting an antioxidant response which could be explained by the modulation of the response from glial cells to dopaminergic neuronal damage induced with 6-OHDA.

## 1. Introduction

The selective degeneration of dopaminergic neurons within the substantia nigra *pars compacta* (SN*pc*) and the consequent denervation of the striatum result in diminished DA levels. This neurochemical deficit manifests as the hallmark motor symptoms of PD [1]. However, the precise neurobiological mechanisms underlying the demise of dopaminergic neurons are multifaceted and remain to be fully elucidated.

Converging lines of evidence implicate oxidative damage and mitochondrial dysfunction, in conjunction with a decline in endogenous antioxidant capacity, as pivotal factors contributing to the damage and eventual death of dopaminergic neurons [2]. Studies involving both PD patients and experimental models of parkinsonism have demonstrated elevated levels of 4-hydroxy-2-nonenal (HNE), increased malondialdehyde levels [3], carbonyl modifications of soluble proteins, auto-oxidation of DA, and increased levels of nucleic acid oxidation products, including 8-OHdG [4]. These findings underscore the significant role of oxidative stress in the pathogenesis of PD.

Furthermore, research suggests an active role for glial cells, particularly the microglia, in the neurodegenerative process affecting dopaminergic neurons. Upon activation, the microglia become a significant source of superoxide and nitric oxide, both of which contribute to the escalation of oxidative stress within the microenvironment of dopaminergic neurons [5]. It is noteworthy that astrocyte-mediated astrogliosis in the SN*pc* has been shown to trigger the activation of the microglia from a resting M0 to a proinflammatory M1 phenotype. Additionally, the infiltration of peripheral immune cells, specifically macrophages (including T cells), into the SN*pc* has been observed, further perpetuating the cycle of dopaminergic cell damage [6, 7].

Clinical and preclinical studies have revealed a compelling correlation between degeneration of dopaminergic neurons, increased oxidative stress, and gut microbiota dysbiosis [8]. This intriguing link suggests a potential role for gut–brain interactions in the pathogenesis of PD. In this context, an overexpression of proinflammatory bacteria has been observed in the intestine of patients with PD [9] and a decrease in TAC; in addition, it has been shown in *in vitro* and *in vivo* studies that *Lactobacillus* exerts neuroprotective effects through the inhibition of dopaminergic cell death and has a potential effect in the management of motor and nonmotor symptoms of PD [10, 11]. In this sense, it has been shown that probiotic supplementation can attenuate oxidative stress and improve total antioxidant capacity in women who suffer from migraines [12]. Furthermore, our research group has previously shown that supplementation with a specific probiotic cocktail (LLH135) of *L. fermentum* LH01, *L. reuteri* LH03, and *L. plantarum* LH05 conferred neuroprotection to dopaminergic cells in a model of hemiparkinsonism. This protective effect was accompanied by improvements in motor coordination, a reduction in the permeability of the blood–brain barrier [13]. It is worth mentioning that this strain has probiotic potential due to its ability to resist low pHs, bile salts, resistance to exposure to gastric enzymes, hydrophobicity capacity, self-aggregation, and antimicrobial activity against different pathogens [14].

However, it remains to be elucidated whether the administration of LLH135 exerts a similar antioxidant effect in a 6-OHDA-induced hemiparkinsonism model. Specifically, further research is warranted to determine if LLH135 can modulate the enzymatic activity of superoxide dismutase, enhance antioxidant capacity, and mitigate DNA oxidation in this model system.

## 2. Materials and Methods

This study was reviewed and approved by the Local Health Research Ethics Committee 1305, under institutional registration number R-2023-1305-035, chaired by Dr. Blanca Miriam de Guadalupe Torres Mendoza, on May 31, 2023.

### 2.1. Probiotic LLH135 Production

The probiotic strains *Limosilactobacillus fermentum* LH01*, Limosilactobacillus reuteri* LH03, and *Lactiplantibacillus plantarum* LH05 were isolated from human breast milk and are part of the culture collection of the “Laboratorio de Investigación Leche humana” at the University of Guadalajara. Following the protocol described by Napoles-Medina et al., the bacteria were cultured and then resuspended in sterile saline solution at a concentration of 10^9^ colony-forming units per milliliter (CFU/mL) for oral administration. The prepared probiotic mixture was stored frozen until use [13].

### 2.2. Animals and Experimental Design

**Figure 1:**
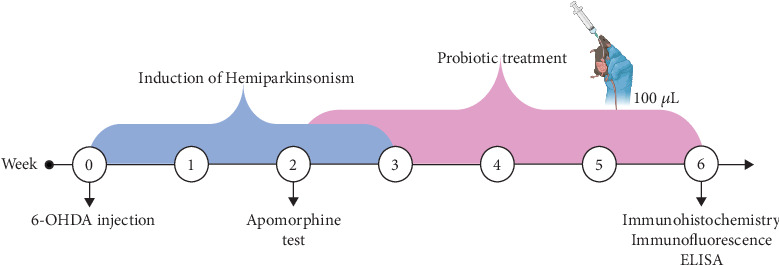
Experimental design of the hemiparkinsonism induction model and the treatment regimen with the probiotic LLH135.

An experimental study was conducted on 48 male C57BL/6 mice (23–27 g) housed under controlled temperature (23 ± 2°C) and a 12:12-h light–dark cycle, with ad libitum access to food and water. All experimental procedures were performed in accordance with the Mexican Official Standard NOM-062-ZOO-1999 for the care and use of laboratory animals. The mice, obtained from the animal facility of the “Centro de Investigación Biomédica de Occidente,” were randomly assigned to four experimental groups (*n* = 12 per group): sham (intrastriatal injection of 2 *μ*L of 6-OHDA vehicle), 6-OHDA+LLH135 (intrastriatal injection of 6-OHDA followed by oral administration of LLH135 for 28 days, starting 14 days after the lesion), and 6-OHDA (intrastriatal injection of 10 *μ*g/2 *μ*L of 6-OHDA) LLH135 (oral administration of 100 *μ*L of the probiotic mixture LLH135 for 28 days) (Figure 1).

### 2.3. 6-OHDA Lesion

All mice received an intraperitoneal injection of 25 mg/kg imipramine (Cat. 28.626-5; Sigma-Aldrich) dissolved in saline 30 min prior to 6-OHDA (Cat. H4381; Sigma-Aldrich) injection to prevent damage to noradrenergic fibers. Mice were anesthetized via continuous inhalation of 1%–2% isoflurane (Fluriso: VetOne 99%). Subsequently, they were placed in a stereotaxic frame to target the injection to the striatum, using coordinates related to Bregma (ML +2 mm, AP +0.5 mm, DV −3.3 mm). Mice received a unilateral injection of 6-OHDA (10 *μ*g in 2 *μ*L of vehicle containing 0.1% ascorbic acid) into the left striatum. The needle was left in place for 5 min to allow for maximal diffusion of the toxin. Immediately after surgery and on the following day, all mice received fluid therapy (5% sterile glucose solution, subcutaneous, 10 mL/kg) to prevent dehydration [15].

### 2.4. Apomorphine Test

Then 2 weeks after surgery, rotational behavior was induced in mice by subcutaneous administration of apomorphine (Cat. 314-19-2; Tocris) at a dose of 0.1 mg/kg. Animals were evaluated for 25 min in a transparent acrylic cylinder (13-cm diameter). The total number of complete rotations contralateral to the lesion was counted, and a minimum of 75 rotations was considered as the inclusion criterion for subsequent tests.

### 2.5. IBA-1, CD68, and GFAP Immunohistochemistry

Following the probiotic treatment period, six animals from each experimental group were transcardially perfused. Brains were surgically removed and cut into 30-*μ*m-thick coronal sections using a vibratome. These sections were then processed for immunohistochemical detection of inflammatory markers in the striatum.

Tissue sections were washed with 1x PBS, and then, epitope unmasking was performed by incubation in sodium citrate buffer (0.3%) for 30 min at 37°C. Endogenous peroxidase activity was blocked with hydrogen peroxide (H_2_O_2_, 10%) for 30 min. Sections were then incubated with 10% fetal bovine serum for 2 h at room temperature to block nonspecific binding sites.

Immunostaining was performed by incubating the sections at 4°C for 24 h with the following primary antibodies: anti-IBA-1 (1:500, PA5-18039, Invitrogen), anti-CD68 (1:500, PA578996, Invitrogen), and anti-GFAP (1:500, sc-33673, Santa Cruz Biotechnology). After washing with 1x PBS, sections were incubated for 2 h at room temperature with the corresponding biotinylated secondary antibodies: goat anti-IgG (for IBA-1, 1:1000, BA-9500, Vector Laboratories), rabbit anti-IgG (for CD68, 1:1000, BA1000, Vector Laboratories), and mouse anti-IgG (for GFAP, 1:1000, BA-2000, Vector Laboratories).

Finally, sections were incubated with an avidin-biotin complex (Cat. PK6100; Vector Laboratories) for 2 h at room temperature and immunoreactivity was visualized using diaminobenzidine (DAB, Cat. D5905; Sigma).

Microglial activation and astrocytic reactivity were assessed by quantifying IBA-1, CD68, and GFAP immunoreactivity, respectively. Photomicrographs were taken at 20x magnification, and the number of immunoreactive cells was manually counted by two observers blinded to the experimental groups. Counts from both observers were averaged for each animal and used for statistical analysis.

### 2.6. IBA-1/CD68 Double Immunofluorescence

To assess the expression of CD68 in IBA-1-positive microglia, double immunofluorescence was performed. Tissue sections were incubated with 0.3% sodium citrate solution for 30 min at 37°C. Subsequently, a 1-h blocking step was performed at room temperature with a PBS solution containing 0.5% Triton X-100 and 10% fetal bovine serum, under constant agitation.

Sections were incubated for 24 h at 4°C with the following primary antibodies: anti-IBA-1 (1:500, PA5-18039, Invitrogen) and anti-CD68 (1:500, PA578996, Invitrogen). After washing with 1x PBS, sections were incubated for 2 h at room temperature with the corresponding fluorescent secondary antibodies: Alexa Fluor 555-conjugated goat anti-IgG (for IBA-1) and Alexa Fluor 488-conjugated rabbit anti-IgG (for CD68), both diluted 1:500.

Finally, tissues were mounted on slides using a mounting medium containing DAPI (4⁣′,6-diamidino-2-phenylindole, Vectashield, H-1200) for nuclear staining. The slides were stored at 4°C in darkness until analysis. Images were acquired using a confocal microscope equipped with a fluorescent light source and bandpass filters at 360, 488, and 594 nm. LEICA LAS X software was used for image acquisition, and ImageJ was used for colocalization analysis of the fluorescent signals.

### 2.7. Total Antioxidant Capacity

Six mice per experimental group were used. Animals were euthanized by decapitation. Brains were extracted and placed in a Petri dish on ice for striatum dissection. Striatal tissue was homogenized on ice using EDTA-free PBS buffer supplemented with a commercially available protease inhibitor cocktail (Complete TM) using an ultrasonic cell disrupter (Virsonic 100). Homogenates were centrifuged at 10,000 *g* for 15 min at 4°C to obtain the supernatant free of cellular debris. Total antioxidant capacity activity was determined spectrophotometrically in the supernatants using a commercially available kit from MyBioSource (MBS2540515), following the manufacturer's instructions, and expressed as units per milligram of protein (U/mg protein).

### 2.8. Superoxide Dismutase Colorimetric Assay

For the determination of superoxide dismutase activity, brain homogenate samples previously prepared for the T-AOC assay were divided into two aliquots. SOD activity was analyzed using a commercially available kit (SOD activity, Cat. ADI-900-157), which facilitates the differentiation and quantification of SOD isoforms present in the samples. Results were expressed as SOD units per milligram of protein.

### 2.9. 8-Hydroxy-2⁣′-Deoxyguanosin ELISA

The concentration 8-OHdG in the striatum was determined by ELISA. DNA was extracted and digested from 10 mg of tissue using the DNeasy Blood & Tissue Kit following the manufacturer's instructions. Subsequently, the concentration of 8-OHdG was quantified using a commercially available ELISA kit (Abcam, USA; Catalog No. AB201734) according to the manufacturer's instructions. Absorbance was measured at 450 nm, with a kit detection range of 0.94–60 ng/mL and a sensitivity of 0.59 ng/mL.

### 2.10. Statistical Analysis

Data were assessed for normality using the Kolmogorov–Smirnov test. A one-way analysis of variance was conducted. Post hoc analyses were performed using Tukey's or Fisher's least significant difference test. Statistical significance was set at *p* ≤ 0.05. All statistical analyses were performed using GraphPad Prism version 8.0.1.

## 3. Results

### 3.1. Supplementation With Probiotic LLH135 Diminishes IBA-1, GFAP, and CD68 Immunoreactivity in Mice Lesioned With 6-OHDA

#### 3.1.1. IBA-1 Immunoreactivity

**Figure 2:**
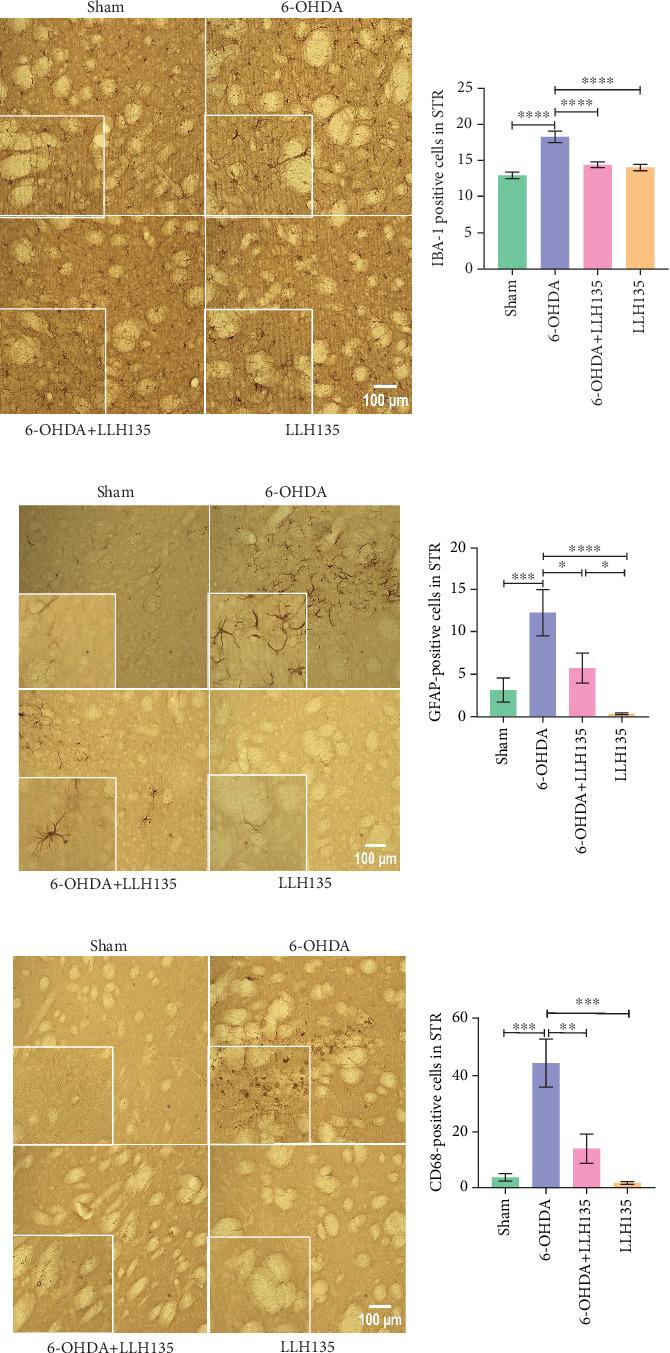
Effect of probiotic LLH135 on the expression of IBA-1 (microglia; a), GFAP (astrocytes; b), and CD68 (microglial cell activation; c). Representative micrographs of immunohistochemistry for IBA-1, GFAP, and CD68 after probiotic LLH135 treatment (4x and 10x magnification). *n* = 6 animals per experiment. Data are presented as mean ± standard deviation. Post hoc analyses were performed using Tukey's honestly significant difference test (IBA-1 and CD68) and Fisher's least significant difference test. ⁣^∗^*p* < 0.05, ⁣^∗∗^*p* < 0.01, ⁣^∗∗∗^*p* < 0.001, and ⁣^∗∗∗∗^*p* < 0.0001.

6-OHDA lesioning induced a significant increase in IBA-1-positive microglia compared to the sham group (*p* < 0.0001). This increase was significantly attenuated by probiotic supplementation following 6-OHDA lesioning (*p* = 0.0001). Probiotic supplementation alone did not significantly alter IBA-1 immunoreactivity compared to the sham group (*p* = 0.4947) but showed a significant reduction compared to the 6-OHDA group (*p* = 0.0001) (Figure 2a).

#### 3.1.2. GFAP Immunoreactivity

GFAP-positive astrocytes were significantly increased in the 6-OHDA group compared to the sham group (*p* = 0.0005). Probiotic treatment following 6-OHDA lesioning significantly reduced the number of GFAP-positive cells compared to the 6-OHDA group (*p* = 0.0112). Similar to the IBA-1 findings, the probiotic-only group showed no significant difference from the sham group but did exhibit significantly reduced GFAP immunoreactivity compared to the 6-OHDA group (*p* < 0.0001) (Figure 2b). The observed changes in GFAP expression are consistent with the known role of astrocytes in response to neuronal injury.

#### 3.1.3. CD68 Immunoreactivity

CD68 immunoreactivity was significantly higher in the 6-OHDA group compared to the sham group (*p* = 0.001). This increase was significantly reduced by probiotic treatment (6-OHDA + LLH135 vs. 6-OHDA, *p* = 0.0028). Consistent with the IBA-1 results, the probiotic-only group “LLH135” did not differ significantly from the sham group but showed a significant reduction compared to the 6-OHDA group (*p* = 0.001) (Figure 2c). CD68, as a microglial marker, indicates microglial activation during inflammation.

#### 3.1.4. Immunofluorescence of IBA-1/CD68 Coexpression

**Figure 3:**
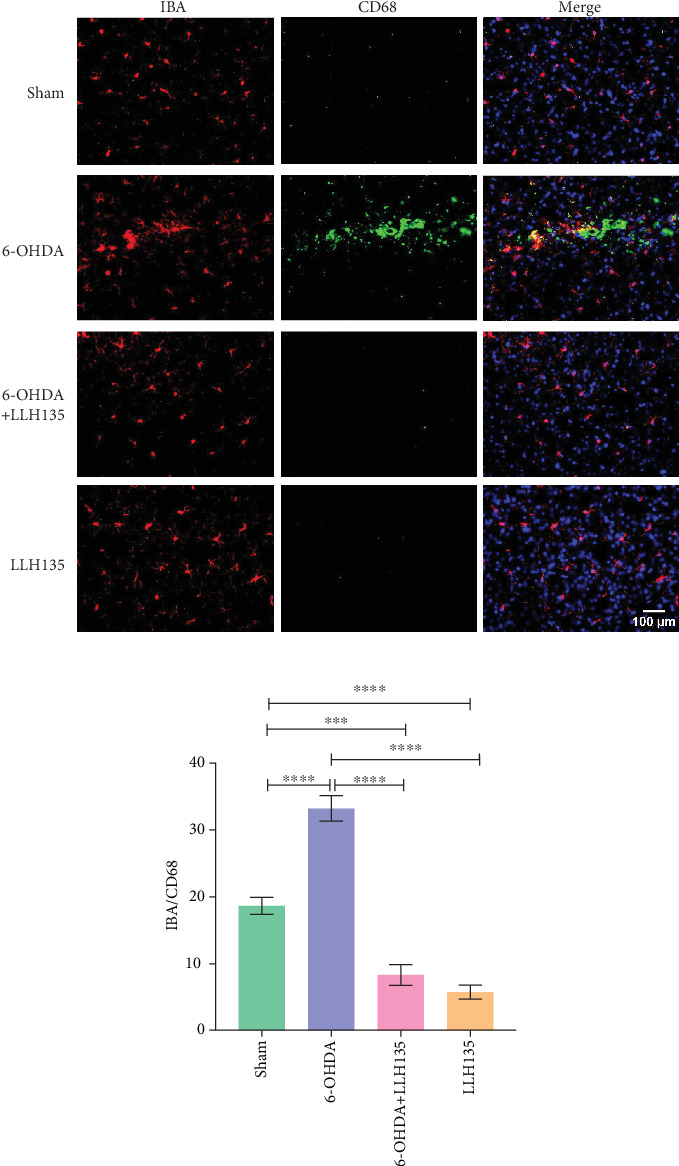
(a) Dual immunofluorescence staining indicates that CD68 (green) was upregulated in animals injured with 6-OHDA, which was labeled with the IBA-1 protein (red). While combined exposure to 6-OHDA+LLH135 downregulation CD68 expression. White arrows indicate CD68/IBA-1 copositive cells. (b) Quantification of CD68/IBA-1-positive cells relative to total IBA-1-positive cells (*n* = 6 animals). Data are presented as mean ± standard deviation. Post hoc analysis was performed using Tukey's test. ⁣^∗∗∗^*p* < 0.001 and ⁣^∗∗∗∗^*p* < 0.0001.

Dual immunofluorescence analysis was conducted to investigate the coexpression of CD68 on microglial cells, utilizing anti-CD68 and anti-IBA-1 antibodies. The findings revealed that 6-OHDA treatment significantly increased the number of cells expressing both markers compared to the sham condition (*p* < 0.0001). Conversely, mice subjected to 6-OHDA injury and subsequently administered the probiotic LLH135 exhibited a reduced number of co-localizing cells with both IBA-1 (red) and CD68 (green) (*p* < 0.0001) (Figure 3).

### 3.2. Probiotic LLH135 Impact on 6-OHDA-Induced TAC

**Figure 4:**
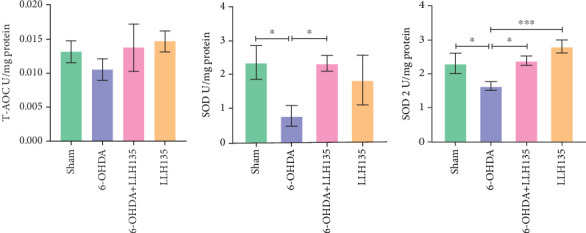
Effects of probiotic LLH135 on the total antioxidant capacity levels (a) and superoxide dismutase enzyme activity (b, c) in 6-OHDA-lesioned animals. Data represents the mean ± standard deviation; ⁣^∗^*p* < 0.05 and ⁣^∗∗∗^*p* < 0.001 (*n* = 6 animals per experiment). Post hoc analysis was performed using Fisher's least significant difference test.

TAC in the striatum did not differ significantly between groups (*F* = 1.08, *p* = 0.3707). However, there was a nonsignificant trend toward decreased TAC in the 6-OHDA group compared to the sham group. Conversely, a nonsignificant trend toward increased TAC was observed in the 6-OHDA group treated with probiotic LLH135 compared to the 6-OHDA group alone (Figure 4a).

### 3.3. The Effect of Probiotic LLH135 on SOD Activity in 6-OHDA-Lesioned Animals

Total SOD activity was significantly decreased in the 6-OHDA group compared to the sham group (*p* = 0.0216). Treatment with the probiotic LLH135 following 6-OHDA lesioning significantly increased total SOD activity compared to the 6-OHDA group alone (*p* = 0.0235) (Figure 4b).

Analysis of the specific activity of the SOD2 isoform revealed a similar pattern. SOD2 activity was significantly decreased in the 6-OHDA group compared to the sham group (*p* = 0.0300). This decrease was reversed in the 6-OHDA group treated with the probiotic LLH135 (*p* = 0.0160) (Figure 4c).

### 3.4. The Effect of Probiotic LLH135 in the Expression of 8-OHdG in 6-OHDA-Lesioned Animals

**Figure 5:**
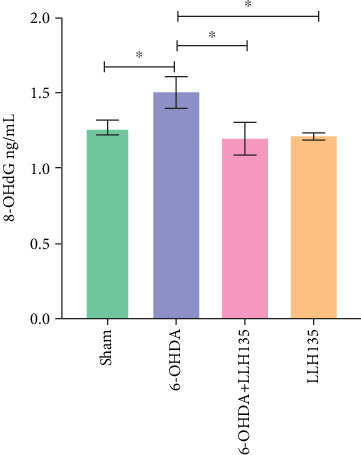
The effect of probiotic LLH135 on the expression of 8-OHdG in 6-OHDA-lesioned animals (*n* = 6 animals). The graph represents the mean ± standard deviation; ⁣^∗^*p* < 0.05. Post hoc analysis was performed using Fisher's least significant difference test.

8-OHdG expression, a marker of DNA oxidation, was significantly increased in the 6-OHDA group compared to the sham group (*p* = 0.044). This increase was significantly attenuated by probiotic LLH135 treatment following 6-OHDA lesioning (*p* = 0.0154). The probiotic-only group “LLH135” did not differ significantly from the sham group (Figure 5).

## 4. Discussion

The precise pathways and mechanisms underlying dopaminergic neuron death in the SN*pc* in PD remain incompletely understood. However, converging evidence implicates increased reactive oxygen species (ROS) generation and diminished antioxidant capacity (SOD, catalase, and glutathione) as central drivers of neurodegeneration. Furthermore, it is known that ROS act on mitochondrial DNA, which is very susceptible to oxidative stress, and there is evidence that ROS produce oxidation of proteins, with their consequent structural deconfiguration. At the lipid level, ROS induce lipid peroxidation that leads to altered cell membrane permeability and corresponding cellular damage and death [16]. Additionally, numerous clinical studies have demonstrated heightened oxidative damage to genetical material coupled with reduced antioxidant capacity among individuals with PD [17]. It is important to note that glial cells (astrocytes and microglia) play an important role in the damage of dopaminergic cells. In this sense, it has been reported that the activation of microglia cells M1 potentiates the death of dopaminergic cells in the population with PD [18], through an increase in the levels of proinflammatory cytokines: tumor necrosis factor-*α* (TNF-*α*), interleukin-6 (IL-6), and interleukin-1*β* (IL-1*β*); also, an increase in the release of ROS [19] and nitric oxide in the cerebrospinal fluid of patients diagnosed with PD [20]. Evidence suggests some probiotics exert neuroprotection via modulation of metal ion levels, such as chelating Fe^++^, which accumulates in microglia during the microgliosis observed in PD [21, 22]. Along with this, one of the primary mechanisms by which microglia facilitates the clearance and phagocytosis of diverse materials and cellular debris is through the production of ROS, mediated by the NADPH oxidase 2 enzyme. This type of protein has the specific function of generating superoxide radicals at the intracellular level and its expression can be activated through the signaling of molecular patterns associated with damage such as the amyloid beta protein in Alzheimer's or the proinflammatory cytokines themselves responsible for signaling its proliferation [23]. However, this is not the only pathway that generates oxidative stress because of the proliferation and activation of this cell line. Recently, it has been shown that ROS from mitochondria also play an important role in the exacerbation and perpetuation of oxidative stress in the face of an insult that produces inflammation [24]. On the other hand, it has been reported that astrocyte activation can be mediated by ROS from NOX2 activity in microglia in various experimental models of parkinsonism induction, to the point that a deletion of the NOX2 gene in these models attenuated astrocyte reactivity [25]. In this way, microglial activation and proliferation precedes astrocytic activation; however, once the latter has been reached, the astrocyte can also contribute to the exacerbation and perpetuation of oxidative stress by producing nitric oxide (NO) [26]. The results of the present work showed an increase in the expression of the DNA oxidative stress marker 8-OHdG, in animals injured with 6-OHDA, in addition to a decrease in the activity of total SOD such as type 2 (SOD2), and this effect is reversed in the group of animals that received the administration of the probiotic LLH135 after injury with 6-OHDA. It is important to highlight that the probiotic LLH135 is composed of a combination of three microbial strains: *Limosilactobacillus fermentum* LH01, *Limosilactobacillus reuteri* LH03, and *Lactiplantibacillus plantarum* LH05. Previous research has reported that *L. plantarum* DP189 exerts a neuroprotective effect through an increase in the expression of nuclear factor erythroid 2-related factor (Nrf2) and its target genes: SOD and glutathione peroxidase (GPx) in a murine model of induction of parkinsonism induced with MPTP. Decrease in the levels of malondialdehyde, ROS, TNF-*α*, IL-6, and IL-1*β* was also observed [27]. Gao et al. reported that the increase in Nrf2 expression is due to the activation of the ERK1/2 signaling pathway. Therefore, *L. plantarum* DP189 can inhibit the expression and release of ROS due to the regulation of the phosphorylation of the ERK1/2 pathway and consequently carry out the activation of the Nrf2 pathway and its binding to the antioxidant response element in DNA and promote the activation of antioxidant genes: SOD, GPx, hemoxygenase 1 (HO-1), and catalase, ultimately preventing the abnormal accumulation of alpha-synuclein (*α*-SYN) and consequently dopaminergic neuronal death and/or damage [27, 28]. On the other hand, *L. fermentum* and *L. reuteri,* the other two strains in the LLH135 blend, have indeed demonstrated beneficial effects relevant to PD. For instance, studies have shown improvements in plasma glutathione levels and a reduction in oxidative stress markers in individuals with PD; these findings suggest that these strains, alongside *L. plantarum*, may contribute synergistically to the overall efficacy of LLH135 [29]. However, the molecular mechanisms underlying these antioxidant effects in parkinsonism models remain to be fully elucidated. Research on ischemia/reperfusion liver injury suggests a potential role for Nrf2/HO-1 pathway activation in mediating the beneficial effects of *Lactobacillus reuteri* [30]. In addition to its antioxidant effects, the probiotic LLH135 demonstrates a multifunctional profile. In this sense, a previous study by our working group demonstrated that this probiotic reverses the motor effects induced by the administration of 6-OHDA, in addition to inhibiting the dopaminergic cell death, lipid peroxidation, and permeability of the blood–brain and intestinal barrier [13]. Now, the results of the present work also demonstrated that this probiotic inhibits the expression and activation of microglia cells, and in the case of astrocytes, only the expression is inhibited in the striatum of animals injured with 6-OHDA. It is important to note the role of microglial cells as the central mediators of the immune response and that their overactivation leads to the loss of dopaminergic neurons in patients with PD [18].

One of the main mechanisms to which the beneficial effect of the administration of probiotics at an antioxidant level in the central nervous system may be due is through the signaling exerted by short-chain fatty acids (SCFAs). It is important to mention that the strains *Lactobacillus reuteri* and *Lactobacillus plantarum* increase the intestinal flora of beneficial bacteria such as *Faecalibacterium prausnitzii*, *Blautia*, *Coprococcus*, *Roseburia*, and *Eubacterium*, which are producers of SCFAs, such as butyrate and propionic acid [31, 32], and that they are decreased in patients with PD [33–37].

Although SCFAs' most relevant concentration is precisely the intestine where the microbiota is found, there are several studies where the participation of monocarboxylate transporters has been verified for its entry into the bloodstream in the intestine and subsequently the expression of these transporters in endothelial cells has also been described, which in this way would allow them to cross the blood–brain barrier [38, 39]. At the glial level, SCFAs exert their neuroprotective effects through the activation of G protein-coupled receptors (GPR41 and GPR43) the now so called Free Fatty Acid Receptors 2 and 3, respectively [40]. At the glial, there is an expression of those receptors and a proposed method by which SCFAs exert their effects is through the inhibition of Histone Deacetylase 1 and 2 (HDAC 1\2), which promotes histone acetylation, favoring the expression of anti-inflammatory genes involved in IL10-STAT3 signaling while suppressing the expression of crucial proinflammatory genes such as TNF-*α* and Stat1, in activated microglia. It is important to mention that HDAC inhibition would serve as a molecular switch that would favor the polarization of microglia cells from a proinflammatory state M1 to an anti-inflammatory state M2, which could mitigate neuroinflammation mediated by glia [41]. Furthermore, it has been reported that butyrate, a SCFA, exerts effects on the redox state, by promoting the activation of the transcription factor NRF2, to subsequently translocate to the nucleus to bind with its antioxidant response element, improving the expression of several antioxidant enzymes (HO-1, n-quinone oxidase 1, GPx, catalase, etc.) and reducing oxidative stress driven by ROS. Furthermore, it has been shown that butyric acid induces epigenetic modifications in the promoter of this transcription factor, activating gene expression of antioxidant enzymes mediated by Nrf2 [42, 43].

Experimental evidence indicates that SCFA can suppress the response of this cellular population. Liu et al. demonstrated that butyrate supplementation exerts neuroprotective effects, by inhibiting neuroinflammation and dopaminergic cell death, through the suppression of microglial activation by modulating the RAS-NFkB signaling pathway in a parkinsonism induction model with MPTP [33]. Furthermore, propionic acid has been shown to be an important fatty acid with immunomodulatory and neuroprotective properties by inhibiting dopaminergic cell death [44] and causing increased neurite outgrowth [45].

The findings of this study indicate that administration of the probiotic LLH135 exerts a neuroprotective effect by mitigating oxidative stress and enhancing overall antioxidant capacity. This suggests the potential utility of LLH135 as an adjunct therapy for PD, particularly in addressing nonmotor symptoms such as constipation. However, further research is warranted to investigate the impact of this probiotic on other nonmotor PD manifestations. Additionally, the existing evidence demonstrates that consumption of the LLH135 probiotic leads to improvements in bowel function among individuals with irritable bowel syndrome, with observable benefits emerging within the first 2 months of intake [46].

## 5. Conclusions

Our findings suggest that the administration of the probiotic LLH135 exerts neuroprotective effects by promoting an antioxidant response by modulating the response of glial cells to dopaminergic neuronal damage with 6-OHDA. All these beneficial effects could be achieved since probiotic LLH135 increases in the intestinal flora of beneficial bacteria which are producers of SCFAs. A limitation of this study is the lack of SCFA quantification and investigation into their interaction with Nrf2, which would further elucidate the neuroprotective mechanisms of LLH135.

## Data Availability

Herein, data back up the findings and any related information is available from the corresponding author upon reasonable request.
